# Oleoylethanolamide and Palmitoylethanolamide Protect Cultured Cortical Neurons Against Hypoxia

**DOI:** 10.1089/can.2018.0013

**Published:** 2018-09-19

**Authors:** Manuel Portavella, Nieves Rodriguez-Espinosa, Pablo Galeano, Eduardo Blanco, Juan I. Romero, Mariana I. Holubiec, Fernando Rodriguez de Fonseca, Emilio Fernández-Espejo

**Affiliations:** ^1^ Laboratory of Animal Behavior and Neuroscience, Department of Experimental Psychology, Faculty of Psychology, Universidad de Sevilla, Seville, Spain.; ^2^ Neurophysiology and Molecular Neurology Lab, Department of Medical Physiology and Biophysics, Faculty of Medicine, Universidad de Sevilla, Seville, Spain.; ^3^ Biochemical Research Institute of Buenos Aires (IIBBA-CONICET), Buenos Aires, Argentina.; ^4^ University of Lleida, Medical Research Institute, Dr. Pifarré Foundation (IRBLleida), Lleida, Spain.; ^5^ UGC Mental Health, Regional University Hospital of Malaga, Institute IBIMA, Málaga, Spain.

**Keywords:** hypoxic–ischemic, neuroprotection, oleoylethanolamide, palmitoylethanolamide, PPARα, TRPV4

## Abstract

**Introduction:** Perinatal hypoxic–ischemic (HI) encephalopathy is defined as a neurological syndrome where the newborn suffers from acute ischemia and hypoxia during the perinatal period. New therapies are needed. The acylethanolamides, oleoylethanolamide (OEA) and palmitoylethanolamide (PEA), possess neuroprotective properties, and they could be effective against perinatal HI. These lipid mediators act through peroxisome proliferator-activated receptors subtype α (PPARα), or transient receptor potential vanilloid (TRPV), such as TRPV subtype 1 and 4.

**Materials and Methods:** The objectives of this study were to discern: (1) the neuroprotective role of OEA and PEA in parietotemporal cortical neurons of newborn rats and mice subjected to hypoxia, and (2) the role of the receptors, PPARα, TRPV1, and TRPV4, in neuroprotective effects. Cell culture of cortical neurons and the lactate dehydrogenase assay was carried out. The role of receptors was discerned by using selective antagonist and agonist ligands, as well as knockout (KO) PPARα mice.

**Results:** The findings indicate that OEA and PEA exert neuroprotective effects on cultured cortical neurons subjected to a hypoxic episode. These protective effects are not mediated by the receptors, PPARα, TRPV1, or TRPV4, because neither PPARα KO mice nor receptor ligands significantly modify OEA and PEA-induced effects. Blocking TRPV4 with RN1734 is neuroprotective *per se*, and cotreatment with OEA and PEA is able to enhance neuroprotective effects of the acylethanolamides. Since stimulating TRPV4 was devoid of effects on OEA and PEA-induced protective effects, effects of RN1734 cotreatment seem to be a consequence of additive actions.

**Conclusion:** The lipid mediators, OEA and PEA, exert neuroprotective effects on cultured cortical neurons subjected to hypoxia. Coadministration of OEA or PEA, and the TRPV4 antagonist RN1734 is able to enhance neuroprotective effects. These *in vitro* results could be of utility for developing new therapeutic tools against perinatal HI.

## Introduction

Perinatal hypoxic–ischemic (HI) encephalopathy is defined as a neurological syndrome where the newborn suffers from acute ischemia and hypoxia during the perinatal period. Brain damage after HI insults is caused by a deleterious combination of glial activation, excitotoxicity, inflammation, and oxidative stress with overproduction of oxidative radicals, such as nitric oxide and reactive oxygen species (ROS)^1–4^ The clinical relevance of this syndrome requires further research in search for protective therapies.

Peroxisome proliferator-activated receptor subtype α (PPARα) is a potential target for neuroprotection against acute ischemia and hypoxia because this receptor plays a prominent role in the modulation of inflammatory and oxidant stress responses.^5–7^ Thus PPARα activation is known to activate astrocytes and glial cells, to reduce the transcription of inflammatory response genes, and to promote neurological recovery by exerting anti-inflammatory effects.^8–15^ Neuroprotective effects of activating PPARα are also associated with a decrease in cerebral oxidative stress.^16^ In this context, there is strong evidence that normal PPARα function is necessary to protect cells from inflammation and oxidative damage in several tissues, such as liver, heart, and spleen.^17^ Furthermore, PPARα agonists have potentially therapeutic efficacy in several neurological diseases, such as Parkinsonism, multiple sclerosis, or autoimmune encephalomyelitis.^18–20^ PPARα can be modulated by lipid mediators, such as the acylethanolamides, oleoylethanolamide (OEA) and palmitoylethanolamide (PEA). These fatty acids act as endogenous ligands for PPARα,^21–23^ and several authors have reported that OEA could exert neuroprotective effects through PPARα.^19,24–26^ Although OEA is an analog of the endocannabinoid anandamide, and PEA enhances anandamide activity, both acylethanolamides do not act directly through cannabinoid (CB) receptors.^21–23,27^

Another family of receptors that are involved in the response to hypoxia is the transient receptor potential vanilloid (TRPV) family. These vanilloid receptors are widely distributed in the central nervous system.^28^ It seems that blocking the transient receptor potential vanilloid subtype 1 or TRPV1 could mediate neuroprotective effects, and OEA-induced neuroprotection could be explained, in part, by its blocking effects on TRPV1.^29–32^ There is also evidence showing that the transient receptor potential vanilloid subtype 4 or TRPV4 is involved in cerebral ischemic–reperfusion injury, and recovery of brain edema.^33–37^ The TRPV4 channel induces an increase in intracellular calcium concentration and plays an important role under physiological and pathological conditions. Blocking TRPV4 inhibits brain edema in cerebral ischemia.^34,35^ TRPV4 channels participate in the pathogenic mechanisms of astroglial reactivity following ischemic insult.^38^

The neuroprotectant role of OEA and PEA in brain damage after acute hypoxia remains to be fully determined. It is worth noting that these compounds might play a physiological role against deleterious effects of hypoxia, because it is known that their brain levels are increased after a traumatic or HI insult.^39–41^ The objectives of this study were to discern: (1) the neuroprotective role of OEA and PEA in cultured cortical neurons subjected to hypoxia, and (2) the role of PPARα, TRPV1, and TRPV4 on possible OEA- and PEA-mediated neuroprotective effects by using selective receptor antagonist and agonist ligands as well as knockout (KO) PPARα mice.

## Materials and Methods

### Animals

Pregnant Wistar rats (250-350 g) from the breeding colony of the University of Seville were used. Pups at P0 were used for *in vitro* experiments. Laboratory temperature was kept at 22±1°C, and a 12-h light/12-h dark cycle (lights on at 08:00 h) was maintained throughout the experiment. Food (laboratory chow) and water were available *ad libitum*.

For further studying the role of PPARα, 1-week-old male *PPARα^+/+^* wild-type (WT) and *PPARα*^−/−^ KO mice were also used. WT and KO C57 BL/6J mice were obtained from Jackson Laboratories (Bar Harbor, ME). KO mice on a C57 BL/6J genetic background were bred in accordance with European Union guidelines for animal care. *PPARα^+/+^* and *PPARα*^−/−^ mice were housed five per cage in temperature—(21±1°C) and humidity (55±10%)—controlled rooms with a 12-h light/12-h dark cycle (light between 08:00 AM and 08:00 PM). Food and water were available *ad libitum* during the whole experiment. Regarding genotyping protocol for *PPARα^+/+^* and *PPARα*^−/−^ mice, it has been described by the authors elsewhere.^42^

### Compounds and protocol

OEA and PEA were purchased from Tocris. OEA and PEA were dissolved in ethanol until use, and they were used for cultures after further dilution at 10% ethanol in Neurobasal, at doses of 0, 5, 10, 20, and 40 μM. The selective PPARα antagonist GW6471 or [(2*S*)-2-[[(1*Z*)-1-Methyl-3-oxo-3-[4-(trifluoromethyl)phenyl]-1-propenyl] amino]-3-[4-[2-(5-methyl-2-phenyl-4-oxazolyl)ethoxyphenylpropyl-carbamic acid ethyl ester was purchased from Tocris, and it was dissolved in 10% dimethyl sulfoxide (DMSO) until use (IC_50_ value of GW6471 is 0.24 μM). The selective antagonist of vanilloid TRPV1 SB 452533 or *N*-(2-Bromophenyl)-N’-[2-[ethyl(3-methylphenyl)amino]ethyl]-urea was purchased from Tocris, and it was dissolved in 10% DMSO until use (pIC_50_=7.0). The selective antagonist of vanilloid TRPV4, RN1734, or 2,4-Dichloro-*N*-isopropyl-*N*-(2-isopropylaminoethyl)benzene sulfonamide, was purchased from Tocris (IC_50_ value of RN1734 is 3.2 μM for rTRPV4). Finally, GSK1016790A, selective agonist of vanilloid TRPV4, was purchased from Sigma-Aldrich. GSK1016790A or (N-((1S)-1-{[4-((2S)-2-{[(2,4-Dichlorophenyl)sulfonyl]amino}-3-hydroxypropanoyl)-1-piperazinyl]carbonyl}-3-methylbutyl)-1-benzothiophene-2-carboxamide) is known to evoke a dose-dependent activation of TRPV4 whole-cell currents at concentrations above 1 nM.

### Cell culture and lactate dehydrogenase assay

Primary cultures of parietotemporal cortical neurons were established as previously described,^43^ with some modifications suggested by others.^44,45^ The cell suspension was adjusted at ∼30,000 cells per well in all tests. Postnatal rat pups (P0) or 1-month-old PPAR-α^*+/+*^ and PPAR-α^−/−^ mice were killed by decapitation, and brains were removed. All animals were humanely sacrificed.

Exposure to pharmacological compounds and hypoxia was initiated after 4 days of *in vitro* conditions.^45^ Treatments were carried out either before or after hypoxia exposure. Regarding experiments before hypoxia, OEA and PEA were added to the culture medium for 30 min, at different concentrations (0, 5, 10, 20, and 40 μM, all compounds). If selective receptor ligands were used (GW6471, SB 452533, RN1734), they were added to the medium 15 min before OEA and PEA. SB 452533 and RN1734 were used at doses of 0, 0.1, 1, 5, and 10 μM. GSK1016790A was used at doses of 1 and 5 nM. In these cases, acylethanolamides were used at the most effective dose, or not added for studying *per se* effects of antagonists. Thereafter, neurons were exposed during 40 min to hypoxia by using hypoxic medium, consisting of Neurobasal without B27, which had been exposed to 95% N_2_/5% CO_2_ air bubbling for 30 min. After this 40-min hypoxia period, the medium was replaced with fresh incubation solution equilibrated with 95% O_2_/5% CO_2_. As regards experiments after hypoxia, the protocol was similar but Neurobasal without B27 was used as the incubation solution equilibrated with 95% O_2_/5% CO_2_, and OEA and PEA were added to the medium for 30 min just after the 40-min hypoxia exposure (antagonists were not used). Times of 30-min incubation of OEA and PEA and 40 min of hypoxia exposure were selected after pilot studies. Thus 30-min incubation was selected because shorter incubation was less effective, and longer incubation did not further modify OEA and PEA-induced effects (data not shown). Forty minutes of hypoxia was selected because it induces 55–65% cell death in cultured cells of rat pups, and 75–85% cell death in cultured cells of mice. These percent values allow better discerning protective effects, because lower values (20 and 30 min of hypoxia) induced lower cell death with higher data variability, and highest values (50 and 60 min of hypoxia) induced strong cell death (data available on request).

After all treatments, the medium was removed, and the cultures were further incubated for 24 h, to carry out the lactate dehydrogenase (LDH) assay.^19^ Cytotoxicity was evaluated by release of the cytosolic enzyme LDH into the culture medium by dying cells (Cytotoxicity Detection Kit; Roche, Indianapolis, IN). Total LDH release or high control was calculated by incubating untreated cells with 0.5% Triton X-100 for 1 h to induce maximal cell lysis. Basal death or low control was calculated from untreated wells with media without B27. Background LDH release was calculated with media alone without cells and the absorbance value obtained from this background control is subtracted from all other absorbance values. The formula of percent cell death was as follows: Percent cell death=[(experimental value-low control)/(high control-low control]×100. Data are given as percent cell survival, which is calculated as (100—percent cell death).

### Statistics and ethics

For statistics, two-way analysis of variance (ANOVA) and *post hoc* tests (one-way ANOVA, Newman–Keuls) were used for statistical comparisons. Regarding OEA and PEA effects on cell survival, two-way ANOVA was used with lipid concentration (0–40 μM) and time of treatment with respect to hypoxia as factors. As regards KO mice, the two variables were lipid concentration (0–40 μM) and genotyping (KO, WT). Finally, when receptor ligands were used, the two factors were lipid coincubation (no, OEA, PEA) and receptor ligand concentration. Experiments were performed according to the animal care guidelines of the European Communities Council (86/609/ECC, 90/679/ECC, 98/81/CEE, 2003/65/EC, Commission Recommendation 2007/526/EC), European Directive 2010/63/EU, and the Spanish Royal Decree 53/2013 on the protection of animals used for research and other scientific purposes. Animal experiments were approved by the local Ethics Committee (CEEA; University of Seville, BIO127).

## Results

### OEA exerts neuroprotection if given either before or after hypoxia

**FIG. 1.**
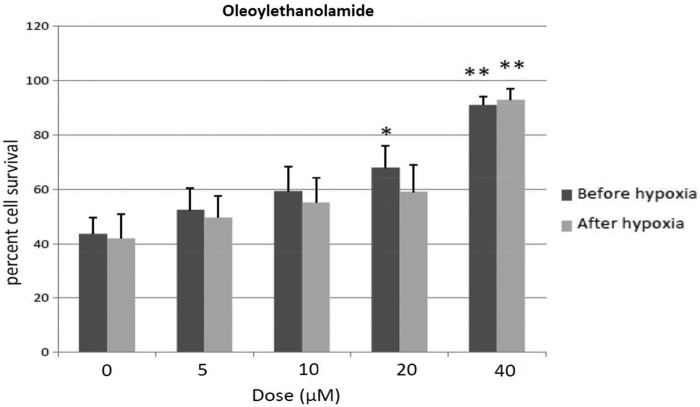
Percent cell survival of cortical neurons after OEA given before and after hypoxia episode. Mean±SEM. *p<0.05, **p<0.01 versus corresponding 0 dose (Newman–Keuls). OEA, oleoylethanolamide; SEM, standard error of the mean.

Two-way ANOVA revealed a dose effect after OEA treatment (F4, 70=11.7, *p*<0.001), without interaction. Thus, OEA given either before or after hypoxia exerted similar effects. One-way ANOVA revealed a significant dose effect after OEA treatment before hypoxia exposure (F4, 44=5.6, *p*<0.01), 20 and 40 μM OEA reliably enhancing cell survival relative to 0 dose-treated cells (20 μM OEA, *p*<0.05; 40 μM OEA, *p*<0.01; Newman–Keuls). If OEA was given after hypoxia, one-way ANOVA revealed a significant dose effect (F4, 39=8.9, *p*<0.01), and *post hoc* analysis indicated that 40 μM OEA exerted a neuroprotective effect (*p*<0.01, Newman–Keuls), as shown in Figure 1.

### PEA exerts neuroprotection if given either before or after hypoxia

**FIG. 2.**
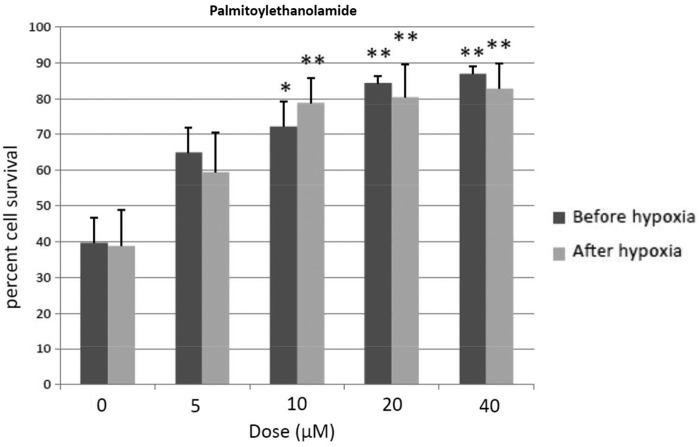
Percent cell survival of cortical neurons after PEA given before and after hypoxia episode. Mean±SEM. *p<0.05, **p<0.01 versus corresponding 0 dose (Newman–Keuls). PEA, palmitoylethanolamide.

Two-way ANOVA revealed a dose effect after PEA treatment (F4, 70=12.6, *p*<0.001), without interaction. Thus PEA given either before or after hypoxia exerted similar effects. One-way ANOVA revealed a significant dose effect after PEA treatment before hypoxia exposure (F4, 44=17.3, *p*<0.001). Thus 10, 20, and 40 μM PEA reliably enhanced cell survival relative to 0 dose-treated cells (10 μM PEA, *p*<0.05; 20 μM and 40 μM PEA, *p*<0.01; Newman–Keuls). If PEA was given after hypoxia, similar changes were found (dose effect, F4, 39=3.2; *p*<0.02; 10, 20 and 40 μM PEA, *p*<0.01; Newman–Keuls), as shown in Figure 2.

### Neuroprotective properties of OEA and PEA are not mediated by PPARα receptors

As explained, to block PPARα, the selective antagonist GW6471 was added to the culture medium. Following GW6471 administration, the survival effects of 40 μM OEA or PEA before hypoxia episode were not significantly modified, as shown in Table 1.

**Table 1.** **Percent Cell Survival Effects of 40 μM Oleoylethanolamide and Palmitoylethanolamide After Adding GW6471, Selective Peroxisome Proliferator-Activated Receptors Subtype α Antagonist**

40 μM OEA	40 μM PEA	GW6471 alone	GW6471 dose (μM)
94.1±3	94.5±3	34.3±3	0
93.3±4	93.6±2	36.1±2	0.1
91.4±2	95.4±4	37.2±4	1
95.2±3	95.3±2	38.3±3	5
93.4±2	96.2±1	38.4±4	10

Mean±SEM. Compounds were added to culture media before hypoxia episode.

OEA, oleoylethanolamide; PEA, palmitoylethanolamide; SEM, standard error of the mean.

**FIG. 3.**
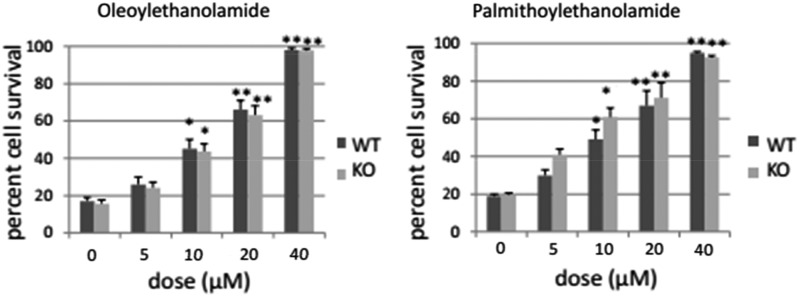
Percent cell survival of cortical neurons after OEA and PEA in *PPARα^+/+^* WT and *PPARα*^−/−^ KO mice. All compounds were given before hypoxia episode. Mean±SEM. *p<0.05, **p<0.01 versus corresponding 0 dose (Newman–Keuls). KO, knockout; PPARα, peroxisome proliferator-activated receptors subtype α; WT, wild-type.

Furthermore, after using *PPARα*^−/−^ mice, the neuroprotective effects of OEA and PEA remain, without significant differences between both groups, as shown in Figure 3. Two-way ANOVA revealed dose effects without interaction for every compound (OEA, F4, 50=155, *p*<0.001; PEA, F4, 50=69, *p*<0.001). Regarding OEA, one-way ANOVA revealed a significant dose effect after OEA treatment before hypoxia exposure (*PPARα*^−/−^ mice, F4, 29=72, *p*<0.001; WT mice, F4, 29=83.3, *p*<0.001), and 10, 20, and 40 μM OEA were observed to significantly enhance cell survival relative to 0 dose-treated cells in both types of cells (10 μM OEA, *p*<0.05; 20 μM OEA, *p*<0.01; 40 μM OEA, *p*<0.01; Newman–Keuls). As regards PEA, one-way ANOVA revealed a significant dose effect after PEA treatment before hypoxia exposure (*PPARα*^−/−^ mice, F4, 29=33.9, *p*<0.001; WT mice, F4, 29=35, *p*<0.001). Thus 10, 20, and 40 μM PEA reliably enhanced cell survival relative to 0 dose-treated cells in both types of cells (10 μM PEA, *p*<0.05; 20 μM and 40 μM PEA, *p*<0.01; Newman–Keuls). Cultured cells of mice seem to better respond to OEA and PEA than those of rats, since 40 μM OEA and PEA enhanced percent cell survival by 75% in cultured neurons of mice, whereas this value was ∼50% in cultured cells of rat pups.

### Neuroprotective properties of OEA and PEA are not mediated by TRPV1

The selective antagonist SB 452533 was given for blocking TRPV1. Following SB 452533 at different doses and 40 μM OEA or PEA, two-way ANOVA did not reveal significant effects, as shown in Table 2.

**Table 2.** **Percent Cell Survival Effects of 40 μM Oleoylethanolamide and Palmitoylethanolamide After Blocking Transient Receptor Potential Vanilloid Subtype 1 with SB 452533**

40 μM OEA	40 μM PEA	SB 45253 alone	SB 452533 dose (μM)
95.2±5	95±3	52.8±4	0
94.4±3	98.1±5	52.6±5	0.1
98.7±4	97.8±2	51.8±5	1
97±5	96.3±2	52±5	5
93±4	95.6±2	54.3±4	10

Mean±SEM. All compounds were given before hypoxia.

### Neuroprotective properties of OEA and PEA are not mediated by TRPV4, and TRPV4 blocking is neuroprotective *per se*

First, the selective agonist GSK1016790A was added to the medium for stimulating TRPV4. GSK1016790A was found to induce full cell death at doses higher than 10 nM (data not shown). Since lower doses induced less cell death, as shown in Table 3, 0, 1, and 5 nM GSK1016790A was used. Following GSK1016790A at 1 and 5 nM, one-way ANOVA revealed a significant dose effect on cell death (F2, 19=12.9, *p*<0.05), as shown in Table 3, because GSK1016790A induced cell death *per se*. Regarding OEA and PEA cotreatment, two-way ANOVA did not reveal interaction or dose effects, because 40 μM OEA or PEA did not induce significant changes in cell death after coadministration with GSK1016790A. Hence, the survival effects of 40 μM OEA or PEA before the hypoxia episode were not significantly modified after GSK1016790A, as shown in Table 3, although this drug induced cell death *per se*, at all the concentrations tested.

**Table 3.** **Percent Cell Survival Effects of 40 μM Oleoylethanolamide and Palmitoylethanolamide After Stimulating or Blocking Transient Receptor Potential Vanilloid Subtype 4 with GSK1016790A or RN1734, Respectively**

Stimulating TRPV4			
40 μM OEA	40 μM PEA	GSK1016790A alone	GSK1016790A (nM)
92.8±4	2.2±3	45.4±6	0
91.2±4	94.1±3	33.4±4^*^	1
93.3±3	92±3	31.5±5^*^	5

Blocking TRPV4			
40 μM OEA	40 μM PEA	RN1734 alone	RN1734 dose (μM)
92.1±3	93.8±4	47.4±5	0
91.2±4	97.1±3	48.3±5	0.1
94.3±4	97±3	55.4±4	1
98±1	100^#^	64.4±3^^^	5
100^#^	100^#^	65.5±5^^^	10

Mean±SEM. All compounds were given before the hypoxia episode.

^*^ *p*<0.05 versus corresponding 0 dose of GSK1016790A alone (Newman–Keuls).

^#^ *p*<0.05 versus corresponding 0 and 0.1 dose of RN1734 + OEA/PEA (Newman–Keuls).

^^^ *p*<0.05 versus corresponding 0 and 0.1 dose of RN1734 alone (Newman–Keuls).

TRPV4, transient receptor potential vanilloid subtype 4.

Second, the selective antagonist RN1734 was added to the medium for blocking TRPV4. RN1734 was neuroprotectant *per se*, as revealed by one-way ANOVA (F4, 24=19.1, *p*<0.05). Thus, the highest RN1734 doses (5 and 10 μM) significantly reduced cell death, as shown in Table 3 (*p*<0.05 vs. the remainder doses, Newman–Keuls). Regarding OEA and PEA cotreatment, two-way ANOVA did not reveal interaction or dose effects. However, following RN1734 at different doses and 40 μM OEA, one-way ANOVA revealed a significant dose effect (F4, 24=24.5, *p*<0.05), as shown in Table 3. The highest RN1734 dose (10 μM) significantly enhanced neuroprotective effects of 40 μM OEA (*p*<0.05 vs. the remainder doses, Newman–Keuls). As regards RN1734 at different doses and 40 μM PEA, one-way ANOVA revealed a significant dose effect (F4, 24=25.2, *p*<0.05). Thus, the highest RN1734 doses (5 and 10 μM) significantly enhanced neuroprotective effects of 40 μM PEA, as shown in Table 3 (*p*<0.05 vs. the remainder doses, Newman–Keuls). Thus, neuroprotective properties of OEA and PEA seem to be enhanced after blocking TRPV4 (Table 3). Since stimulating TRPV4 was devoid of effects on OEA and PEA protective effects, effects of RN1734 cotreatment seem to be a consequence of additive actions.

## Discussion

Brain damage after HI insults is caused by the deleterious combination of glial activation, excitotoxicity, inflammation, and oxidative stress with overproduction of ROS.^1–4^ We have tested in a culturing cell model of neuronal hypoxia some lipid mediators that are known to act through receptors, which are otherwise involved in post-HI reactions. These receptors encompass PPARα, and the vanilloid receptors TRPV1 and TRPV4. These lipid mediators belong to the family of acylethanolamides, which are CB derivatives that do not act through CB receptors.^21–23,27^

The findings of the present study indicate that the fatty acid acylethanolamides, OEA and PEA, exert neuroprotective effects on cultured parietotemporal cortical neurons subjected to a hypoxic episode. These effects are dose dependent, the 40 μM OEA/PEA dose exerting the strongest neuroprotective effects. Contrary to expectation, these effects are not mediated by PPARα, TRPV1, or TRPV4. In this context, these fatty acids exert neuroprotective effects in other cellular and animal models through these receptors,^21–23,29,30^ and OEA and PEA act as endogenous ligands for PPARα, exerting neuroprotective effects.^19–24,26^ OEA is also known to act as a neuroprotectant through the TRPV1 in other situations.^31,32^ To sum up, neuroprotective effects on cortical neurons against acute hypoxia are mediated by mechanisms other than PPARα or vanilloid receptors, TRPV1 or TRPV4.

Interestingly, blocking TRPV4 is neuroprotective *per se*. The neuroprotective role of TRPV4 in ischemia episodes has been highlighted recently, because TRPV4 is involved in cerebral ischemic–reperfusion injury and recovery of brain edema.^33,34^ Stimulation of TRPV4 is observed to be cytotoxic *per se* for cultured cortical neurons, as expected since the activation of TRPV4 is known to induce cytotoxicity in many types of cells.^34–37^

It is of note that TRPV4 blocking reliably enhances the neuroprotective effects of OEA and PEA. Since TRPV4 activation is devoid of effects on neuroprotection of both acylethanolamides, the findings after TRPV4 blocking could be accounted for by additive actions rather than pharmacodynamic interactions between coadministered drugs. It is worth noting that a complete protection against hypoxia of cortical neurons is afforded by high doses of the TRPV4 antagonist RN1734 when it is coadministered with 40 μM OEA or PEA. The additive action of TRPV4 blocking could be explained by diverse mechanisms. Thus, TRPV4 channels participate in changes in intracellular calcium concentration and astroglial reactivity, and TRPV4 are upregulated during cerebral ischemia.^34–36,38^ TRPV4 is also known to be sensitive to cell swelling and arachidonic acid and its metabolites, which are associated with cerebral ischemia.^35^ These hypoxia-induced deleterious effects might be further attenuated after blocking vanilloid TRPV4. Finally, TRPV4 blocking inhibits brain edema in cerebral ischemia, and reactive astrocytosis after stroke.^34,37^

The results of the present study point to new therapeutic approaches for fighting cortical neuron death after a hypoxia episode, because treatment with the lipids OEA and PEA or cotreatment with these acylethanolamides and TRPV4 antagonists might be pharmacological tools with protective effects *in vivo* as do they *in vitro*.

## Abbreviations Used

ANOVA: analysis of variance
CB: cannabinoid
DMSO: dimethyl sulfoxide
HI: hypoxic–ischemic
KO: knockout
LDH: lactate dehydrogenase
OEA: oleoylethanolamide
PEA: palmitoylethanolamide
PPARα: peroxisome proliferator-activated receptors subtype α
ROS: reactive oxygen species
TRPV: transient receptor potential vanilloid
WT: wild-type
